# The incommensurate composite Y_*x*_Os_4_B_4_ (*x* = 1.161)

**DOI:** 10.1107/S205252062400982X

**Published:** 2024-10-31

**Authors:** Berthold Stöger, Oksana Sologub, Leonid Salamakha

**Affiliations:** a X-Ray Centre TU Wien Getreidemarkt 9 1060Vienna Austria; b Institute of Solid State Physics TU Wien Wiedner Hauptstraße 8–10 1040Vienna Austria; SIMaP, France

**Keywords:** borides, superconducting alloys, incommensurate composites, superspace approach

## Abstract

Y_*x*_Os_4_B_4_ (*x* = 1.161) crystallizes as a tetragonal incommensurate composite of columns of Y atoms extending along [001] in an Os_4_B_4_ framework. The structure was refined using the superspace approach. The basic structure of the Y subsystem can be idealized as having *I*4/*mmm* symmetry, with a crystallographically unique Y atom located on the 4/*mmm* position. The actual superspace symmetry is *P*4_2_/*nmc*(00σ_3_)*s*0*s*0. The Y atoms feature only subtle positional modulation in the [001] direction. The Os_4_B_4_ subsystem [*P*4_2_/*ncm*(00σ_3_)00*ss* superspace symmetry] is built of columns of edge-sharing Os_4_ tetrahedra extending along [001] and B_2_ dumbbells. The Os_4_ tetrahedra feature pronounced positional modulation with a distinct variation of the Os—Os bond lengths. Modulation of the B_2_ dumbbells is best described as a rotation about the [001] axis.

## Abstract 2

Tetragonal Y_*x*_Os_4_B_4_ (*x* = 1.161) is an incommensurate composite of columns of Y atoms in a three-dimensional Os_4_B_4_ framework. The structure was refined using the superspace approach.

## 1. Introduction

The borides *RM*_4_B_4_ (*R* = rare earth metal, *M* = transition metal) constitute a family of compounds exhibiting a rich variety of interesting physical properties (superconductivity, ferromagnetism, re-entrant superconductivity) (Johnston & Braun, 1982 ▸; Maple *et al.*, 1982 ▸). Depending on the *R* and *M* constituents, these compounds crystallize in four structure types, CeCo_4_B_4_ (space group *P*4_2_/*nmc*) (Ku’zma & Bilonizhko, 1972 ▸), LuRu_4_B_4_ (space group *I*4_1_/*acd*) (Johnston, 1977 ▸), LuRh_4_B_4_ (space group *Ccca*) (Yvon & Johnston, 1982 ▸) and NdCo_4_B_4_ (space group *P*4_2_/*n*) (Ku’zma & Bilonizhko, 1978 ▸), which can be considered as a family of polytypes (Yvon & Johnston, 1982 ▸). The common structural units of all these structures are B—B dumbbells, *M*_4_ tetrahedra and columns of *R* atoms.

Despite the spectacular properties of reported *RM*_4_B_4_ compounds, the borides with *M* = Os have not been studied sufficiently. The NdCo_4_B_4_ structure was proved for *R*Os_4_B_4_ (*R* = La–Nd, Sm); a different but unknown structural variant has been suggested for compounds of 1–4–4 stoichiometry occurring in the *R*–Os–B system when *R* is a heavy rare earth element or yttrium (Rogl, 1979 ▸; Ku, 1980 ▸).

In continuation of our studies of the Y–Os–B system (Sologub *et al.*, 2007 ▸), we explored the B-rich corner and obtained phases with composition Y_*x*_Os_4_B_4_ with *x* close to one. Careful examination of single-crystal X-ray diffraction data revealed the structural complexities of this compound, reminiscent of those found in related boride systems with other transition metals (Zavalij *et al.*, 1994 ▸; Bezinge *et al.*, 1985 ▸).

Structurally, Y_*x*_Os_4_B_4_ belongs to a family of compounds with a structure derived from NdCo_4_B_4_. These *R*_*x*_*M*_4_B_4_ compounds crystallize in tetragonal commensurate or incommensurate composite structures (van Smaalen, 2007 ▸) of *R* columns contained in an *M*_4_B_4_ framework. The incommensurability of Y_*x*_Os_4_B_4_ has been observed by Zavalij *et al.* (1994 ▸) but no structure refinement has been published up to now. Other known members of the family are various iron borides *R*_*x*_(Fe_4_B_4_) (Bezinge *et al.*, 1985 ▸) and the manganese borides Pr_*x*_(Mn_4_B_4_) and Pr_*x*_(Re_4_B_4_) (Zavalij *et al.*, 1994 ▸).

To the best of our knowledge, refinements of these structures have always been restricted to either individual sub­systems or to commensurate approximants. Here, we report on the synthesis of Y_*x*_Os_4_B_4_ and the refinement of its incommensurate structure using the superspace approach (Wolff *et al.*, 1981 ▸).

## 2. Experimental

### 2.1. Synthesis

A sample with a total weight of 0.5 g was synthesized by arc melting appropriate amounts of the constituent elements in stoichiometry ∼1:4:4 under a Ti-gettered high-purity argon atmosphere on a water-cooled copper hearth. Pieces of yttrium (ChemPur, Germany, 99.9 mass%), crystalline boron (ChemPur, Germany, 99.8 mass%) and re-melted pellets of compacted osmium powder (Sigma–Aldrich, USA, 99.9 mass%) were used as starting materials. The arc-melted button was cut into pieces, from which a larger portion of alloy was wrapped in tantalum foil and vacuum-sealed in a quartz tube for annealing at 800°C for 240 h. Crystals were isolated via mechanical fragmentation of the annealed alloy. Single-crystal X-ray intensity data were collected for the specimen of best quality, which was verified via preliminary inspection on a four-circle Bruker APEX II diffractometer (CCD detector, κ geometry, Mo *K*α radiation).

### 2.2. Single-crystal diffraction

Intensity data from a tiny crystal of the title compound were collected at 300 K in a dry stream of nitrogen on a Stoe STADIVARI diffractometer system equipped with a Mo *K*α micro-source and a DECTRIS Eiger CdTe hybrid photon-counting (HPC) detector. Data were processed using *X-AREA* (Stoe & Cie GmbH, 2021 ▸). Data reduction was performed as a 3+1-dimensional modulated structure with satellites up to the second order. Corrections for absorption effects were applied using the multi-scan approach followed by a spherical absorption correction implemented in *LANA* (Stoe & Cie GmbH, 2021 ▸). An initial model was derived from the published data of related boride phases. The structure was refined with *JANA2006* (Petříček *et al.*, 2014 ▸). Data collection and refinement details are compiled in Table 1 ▸ and an overview of the cell parameters and symmetries of the two subsystems is given in Table 2 ▸.

## 3. Results and discussion

### 3.1. Indexing of the diffraction pattern

Y_*x*_Os_4_B_4_ is an incommensurate composite of two tetragonal subsystems, Os_4_O_4_ and Y. Let 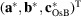 and 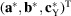 be the reciprocal bases of these two subsystems, which share the a* and b* basis vectors. Note that, by convention, we write reciprocal bases as columns, as indicated by the superscript T, since they transform as *contravariant* tensors (Sands, 2002 ▸). In a classical treatment of an incommensurate composite, the reflections would be indexed using the four-dimensional basis 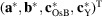. The first three basis vectors correspond to the reciprocal basis of Os_4_B_4_, which is used as the ‘reference system’ because it contributes significantly more to the diffraction intensity than the Y subsystem.

**Figure 1:**
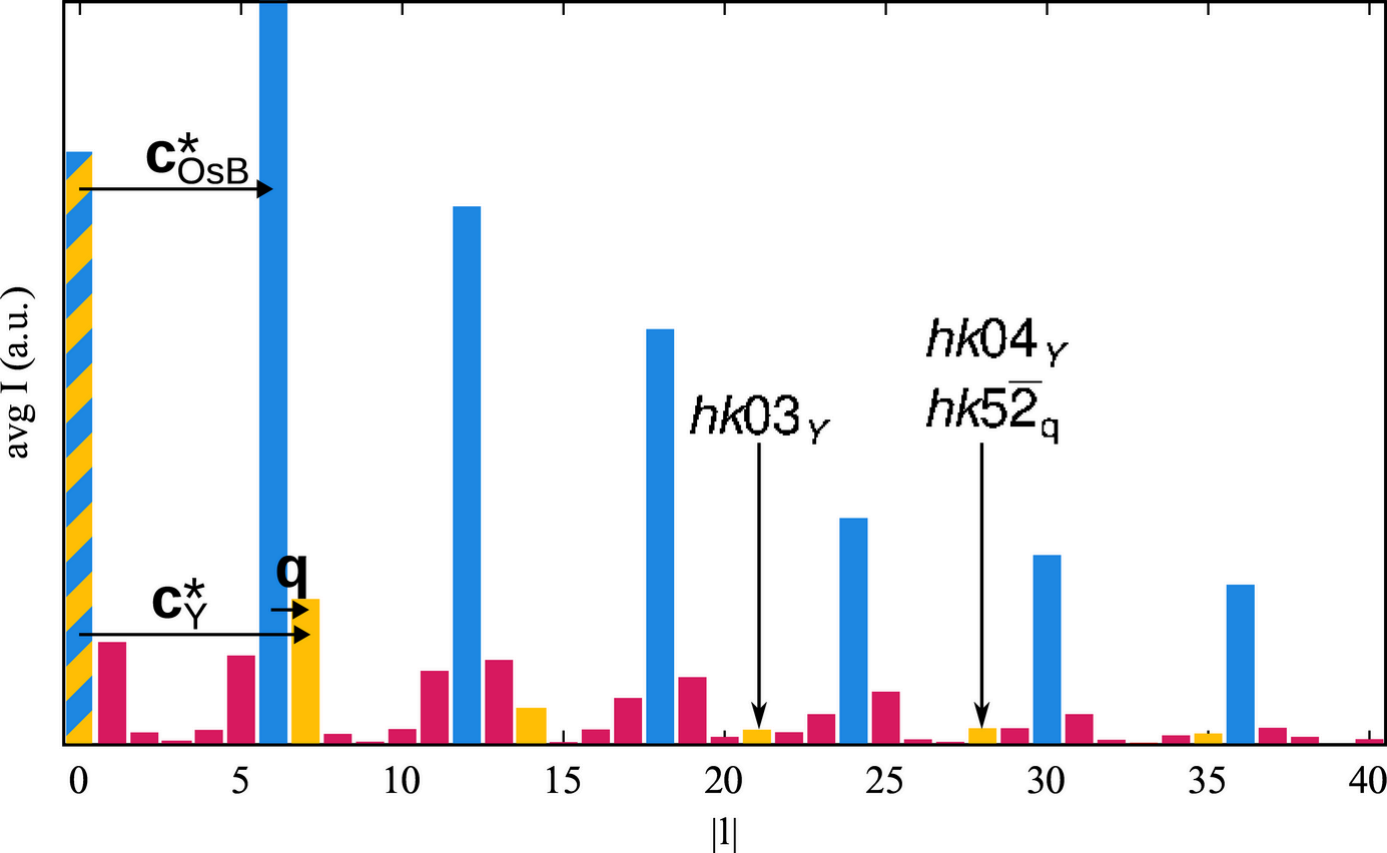
Average reflection intensities plotted versus |*l*| when integrated as a sixfold (Os_4_B_4_) or sevenfold (Y) superstructure. Yellow (Os_4_B_4_) and blue (Y) indicate main reflections of the corresponding subsystem. The |*l*| = 21 and |*l*| = 28 reflections discussed in the text are marked by arrows.

The satellite order of the *hklm*_Y_ reflection (subscript Y since 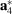 = 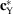) is then given by 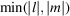. However, the observed diffraction intensities suggested a different integration approach. Since *c*_OsB_/*c*_Y_ ≃ 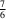, the structure can be approximated by a sixfold superstructure with respect to the Os_4_B_4_ subsystem, or sevenfold with respect to the Y subsystem. Fig. 1 ▸ gives the average reflection intensity by |*l*| when integrated in such a super-cell setting. The main reflections of the Os_4_B_4_ subsystem are marked in blue and those of the Y subsystem in yellow. The figure shows that |*l*| = 6*n* ± 1 reflections, which are located next to the Os_4_B_4_ subsystem’s main reflections (|*l*| = 6*n*), are in general stronger than the Y subsystem’s main reflections (|*l*| = 7*n*).

Therefore, we applied a change of reciprocal basis to 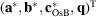 with 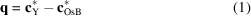
according to the following scheme.
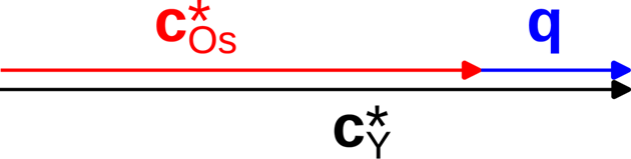


Expressed with respect to 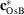, we obtain **q** = 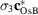, with σ_3_ = 0.16134 (8) ≃ 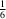. We used this cell and modulation vector and integrated with satellites up to the second order. The residuals listed in Table 1 ▸ are given with respect to this satellite order. Reflection indices with respect to this basis will be given with a subscript *q*.

Note that the two indexing approaches are in fact different. For example, the *l* = 28 superstructure reflections are *hk*04_Y_ main reflections with respect to the 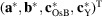 basis or 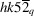 second-order satellites with respect to 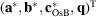. These are indeed different reflections in reciprocal superspace: when transforming 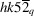 back, one obtains the indexes 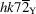, *i.e.* still a second-order satellite. Since the intensity of this reflection is dominated by the second-order satellite, it is reasonable to integrate using **q** as the modulation wavevector.

This highlights the fundamental problem of reflection overlap: *hk*04_Y_ and 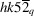 will both contribute to the integrated reflection intensity. However, the *JANA2006* software can detect close reflections and will treat these intensities as the sum of two reflections. From a refinement and symmetry point of view, these two settings are therefore equivalent.

Likewise, the weak reflections halfway between the Os_4_B_4_ main reflections (*hkl*3_*q*_ and 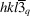) could not be properly resolved into the *m* = 3 and *m* = −3 components. When including these reflections in the integration, the reliability factors of the refinements (and also of the main and low-order satellite reflections) worsened. An integration using the sixfold supercell (with respect to the Os_4_Y_4_ system) and transformation into the incommensurate cell likewise led to distinctly worsened reliability factors. Ultimately, we therefore only used satellites up to the second order. This means that the *hk*03_Y_ and 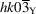 main reflections of the Y subsystem were not included in the refinements (see Fig. 1 ▸). However, this appears acceptable, given all the other Y subsystem main reflections are included (even if indirectly).

The reflection overlap likewise affects the evaluation of the length of the **q** vector and consequently the evaluation of the chemical composition. Different lengths were obtained from different data reduction strategies (*e.g.* different maximum satellite order or independent integration of both subsystems).

The length given here was derived from the integration used for the refinements (with satellites up to the second order). It has to be stressed, however, that the actual uncertainty in the length of **q** is larger than that determined by the integration software. In all integration attempts, σ_3_ was distinctly smaller than 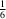 and inspection of the images showed splitting or enlargement of satellites in the c* direction, confirming the incommensurate character of the structure.

### 3.2. Superspace embedding

Each subsystem of an incommensurate composite is modulated with a modulation wave corresponding to the periodicity of the other subsystem(s). Thus, it is useful to embed the structure in superspace by analogy with classical incommensurately modulated structures. The superspace then has a 3+*d*-dimensional (here *d* = 1) superspace group symmetry. However, as noted by van Smaalen (1991 ▸) and Zeiner & Janssen (2003 ▸), the peculiar situation arises that, when transforming from one subsystem to the other, *non-equivalent* superspace groups may be obtained. In fact, in Y_*x*_Os_4_B_4_, the Os_4_B_4_ subsystem has *P*4_2_/*ncm*(00σ_3_)00*ss* and the Y subsystem has *P*4_2_/*nmc*(00σ_3_)*s*0*s*0 superspace group symmetry (note the interchanging of the direction of the *n* glides and the *m* reflections). The origin of the Y subsystem is moved by 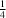 in the *x*_4_ direction with respect to the Os_4_B_4_ subsystem.

**Figure 2:**
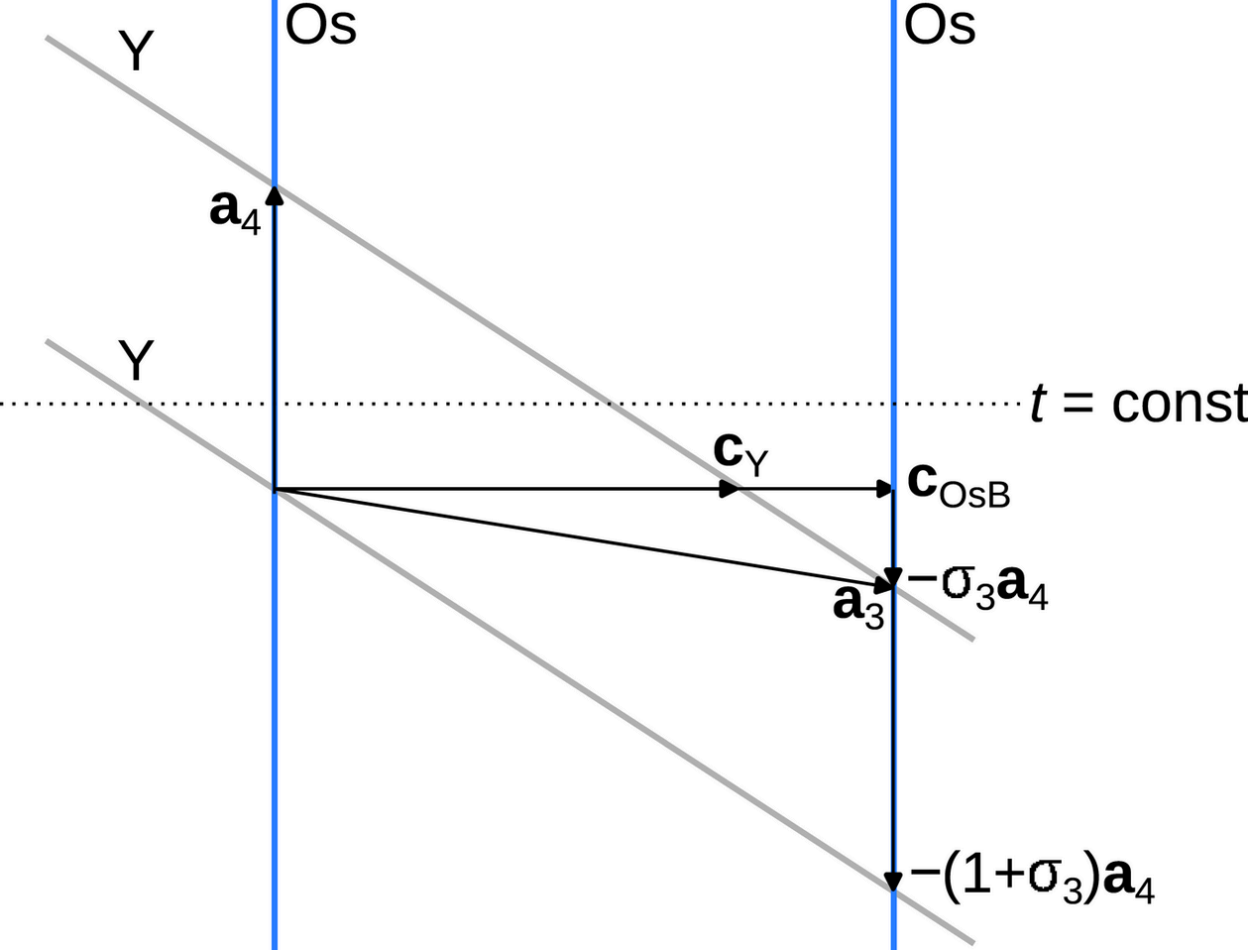
A diagram of the superspace embedding of Y_*x*_Os_4_B_4_ in superspace without positional modulation. The Y (gray) and Os (blue) atoms are represented by lines projected on the (*x*_3_, *x*_4_) plane.

Fig. 2 ▸ schematizes (not to scale) an (*x*_3_, *x*_4_) section of superspace of such a refinement. In particular, it shows the embedding of a Y atom (gray lines) and an Os atom (blue lines), assuming the absence of positional modulation and point-shaped atoms. The real-space structure is given by a *t* = const section perpendicular to **a**_4_, as indicated by a dotted line. The Os lines, since they belong to the ‘reference system’, are parallel to **a**_4_ and spaced by *c*_OsB_. The Y line is inclined in such a way that the Y atoms in a *t* = const section are spaced by *c*_Y_.

The relative slope of the Y line is −(1 + σ_3_). Note that traditionally one would use the vector **c**_OsB_ − (1 + σ_3_)**a**_4_ as the third basis vector **a**_3_, since then the Y lines extend parallel to **a**_3_. Here we chose **a**_3_ = **c**_OsB_ − σ_3_**a**_4_, since this is the dual basis of the reciprocal basis used for integration (Section 3.1). In a sense, this is the reduced supercell, as the angle between **a**_3_ and **a**_1_, **a**_2_ is minimized, resulting in less-skewed unit cells in superspace sections.

The slope of the Y lines is automatically implemented by the *JANA2006* software when relating the reciprocal basis of the Y subsystem 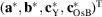 to the refinement basis 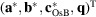 using the **W** matrix. From equation (1) it follows that 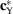 = 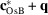. Thus, the basis of the Y subsystem is given as 
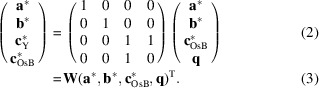


### 3.3. Basic structures

**Figure 3:**
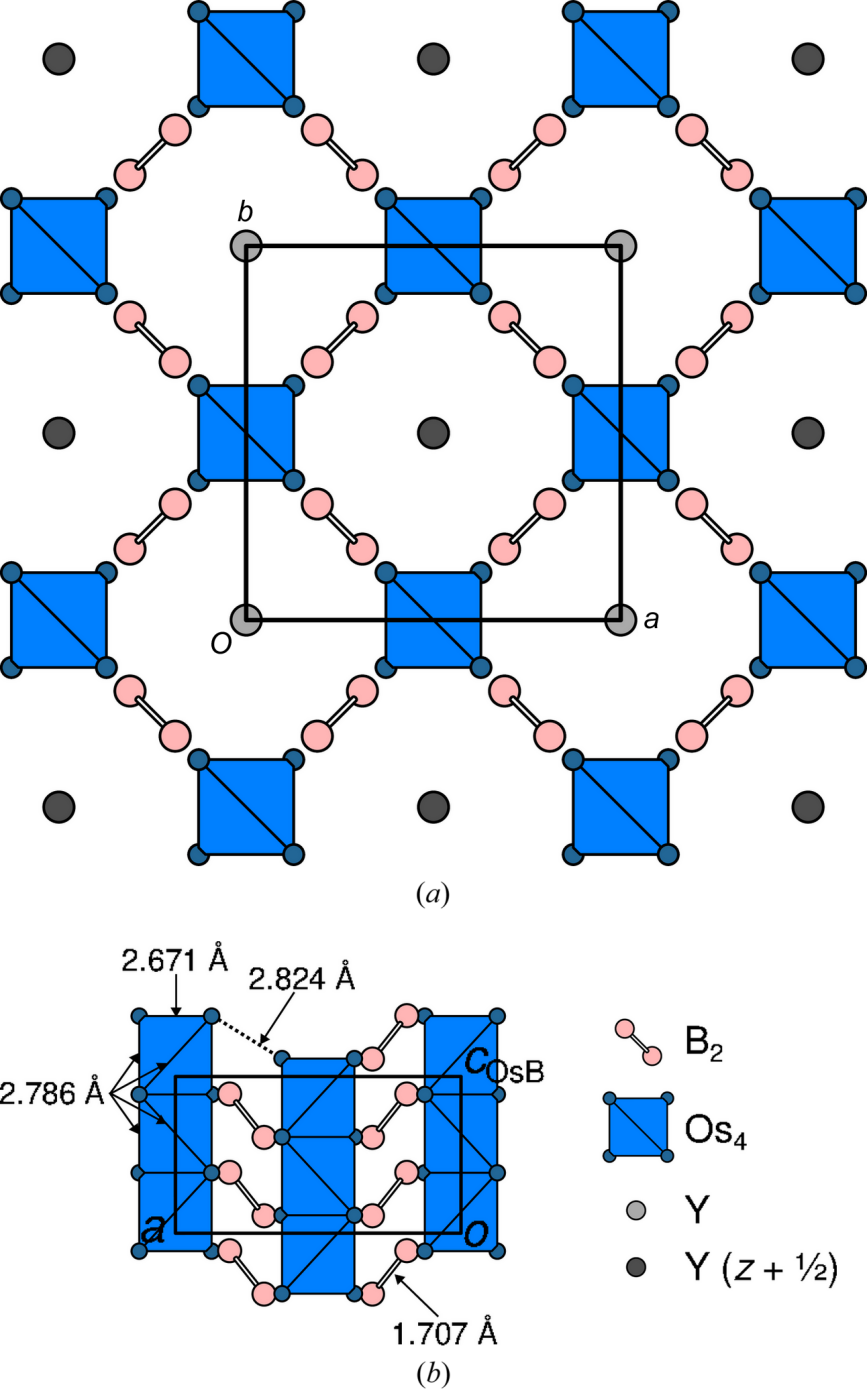
The *P*4_2_/*ncm* basic structure of the Os_4_B_4_ subsystem viewed (*a*) down [001] and (*b*) down [100]. Os_4_ units are represented by blue tetrahedra and B atoms by pink spheres of arbitrary radius. The positions of the Y atoms in the Y subsystem are indicated by gray spheres in panel (*a*).

The two tetragonal subsystems share the **a** and **b** lattice basis vectors [*a* = *b* = 7.4495 (3) Å]. The space-group symmetries of the basic structures are obtained by ignoring the *x*_4_ component of the respective superspace symmetry. Accordingly, the basic structure of the Os_4_B_4_ subsystem [*Z* = 2, *c*_OsB_ = 4.09669 (15) Å] features *P*4_2_/*ncm* symmetry (we use origin choice 1, the origin in 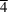). It comprises one crystallographically unique Os and one B atom, both located on the 8*i* Wyckoff position, *i.e* on the *m*_〈110〉_ reflection planes. The Os atoms form columns of edge-sharing Os_4_ tetrahedra, generated by the 4_2_ screw rotations (Fig. 3 ▸). The shared edge represents the shortest Os—Os distance [2.6713 (3) Å] of the structure. Four symmetry-equivalent Os—Os contacts [2.7863 (4) Å] form the non-shared edges of the tetrahedra. The rods are connected by 2.8240 (3) Å Os—Os bonds [dotted line in Fig. 3 ▸(*b*)], whereby these bonds are centered on 2_〈110〉_ rotation axes. The B atoms form B_2_ dumbbells [B—B bond length of 1.707 (8) Å] located on ..2/*m* positions (Fig. 3 ▸). Note that the given geometric parameters were derived by refining against the main reflection of the Os_4_B_4_ subsystem, which technically results in the *average* structure, not the *basic* structure.

The Y subsystem (*Z* = 2, *c*_Y_ = 3.5276 Å) is built of columns of Y atoms centered in the channels of the Os_4_B_4_ subsystem at *x* = *y* = 0 [Fig. 3 ▸(*a*)]. The basic structure has *P*4_2_/*nmc* symmetry (origin choice 1 in 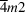). Note again that this is a different space-group type from the OsB subsystem, with the directions of the *c* and *m* reflections inverted. One crystallographically unique *Y* molecule is located on the 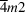 position at *x* = *y* = *z* = 0.

The orbit of an atom is the set of all atoms generated by application of the space group symmetry. If, assuming spherical atoms, this set features higher symmetry than the space group, the orbit is non-characteristic (Engel *et al.*, 1984 ▸). The orbit of the Y atom (located on the 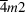 position of *P*4_2_/*nmc*) is non-characteristic with *I*4/*mmm* symmetry. Thus, if the Y atoms are considered as point charges or centrosymmetric electron distributions, the basic structure of the Y subsystem possesses *I*4/*mmm* symmetry, as has been reported earlier (Zavalij *et al.*, 1994 ▸). Due to the *I* centering, every second Y column is translated by **c**_Y_/2 in a checkerboard pattern, as indicated by darker shading in Fig. 3 ▸(*a*).

However, the interaction with the Os_4_B_4_ subsystem must not be neglected and the actual time- and space-averaged electron density of the Y position might not be centro­symmetric. This can be expressed by anharmonic atomic displacement parameters (ADPs). For the sake of argument, we might assume that the averaged electron density is tetrahedral in shape, which is compatible with the 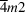 site symmetry. The Y rods at *x* = *y* = 0 and *x* = *y* = 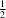 are then inverted with respect to [001] and not related by a translation, *i.e.* the *I* centering is lost. A similar argument can be made for the positional modulation of the Y atom.

The chemical composition of Y_*x*_Os_4_B_4_ is determined by the fraction of the unit-cell volumes of the basic structures of the subsystems: *x* = *c*_OsB_/*c*_Y_ = 1.161 ≃ 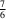.

### 3.4. Modulation of the Y subsystem

According to the superspace group symmetry, the Y atom may be positionally modulated along the *c* direction of the basic structure. No positional modulation is possible in the (*a*, *b*) plane. Since we expected at least a slight influence of the Os_4_B_4_ subsystem on the Y subsystem, the modulation of the *x*_3_ coordinate was modeled with harmonics up to the fourth order. Although such high-order harmonics might seem excessive for a data set with satellites up to the second order, they correspond to merely two modulation parameters, since only odd harmonics of even order [sin(2*n*2π*t*_Y_), 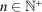] are allowed. Note that the argument to the modulation function depends only on *t*_Y_, because Y is located at the origin of the basic structure. The subscript indicates that *t*_Y_ is given with respect to the Y system. In the remainder of this work *t* will be given with respect to the Os_4_B_4_ system.

**Figure 4:**
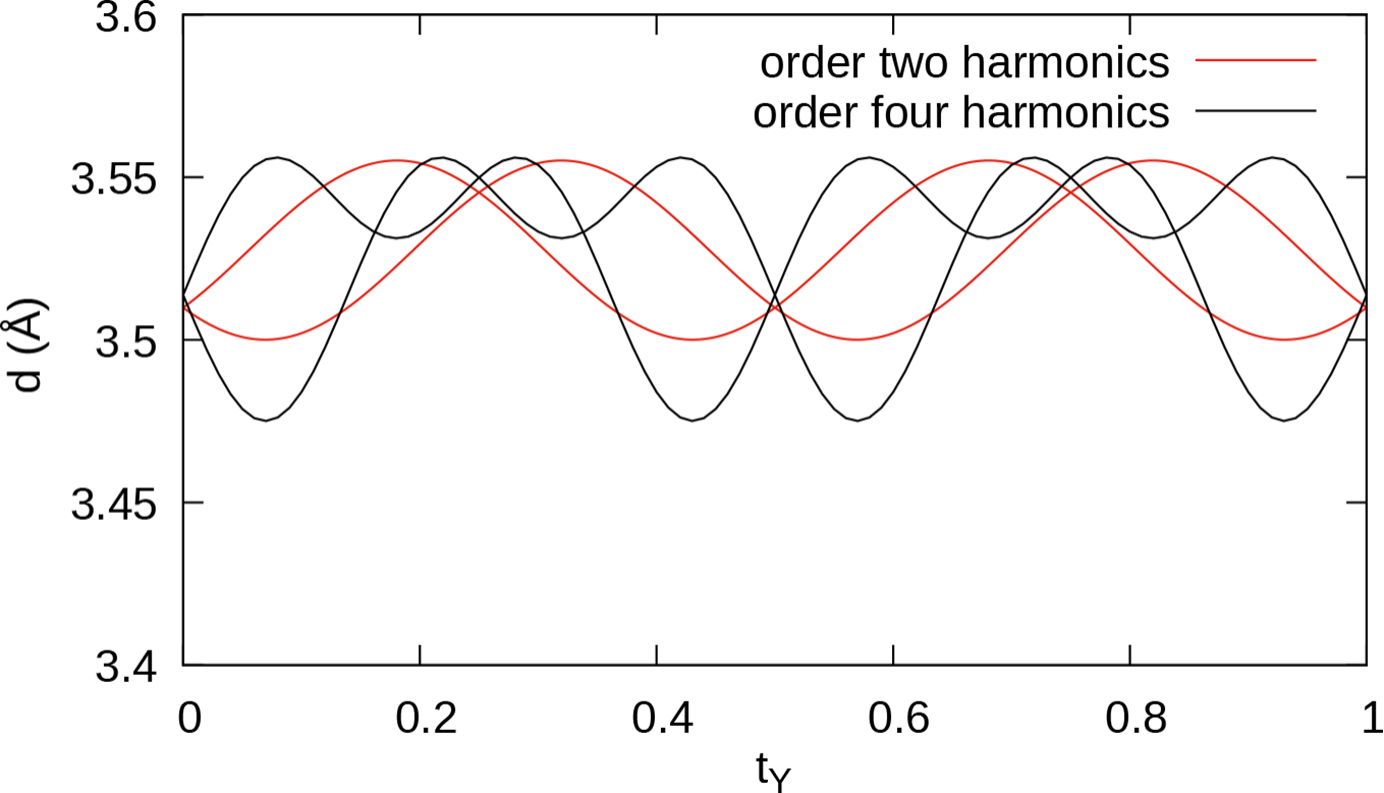
A *t* plot of Y—Y distances when modeling modulation of the *z* coordinate of Y with up to (red) second-order and (black) fourth-order harmonics. Here, in contrast to all other *t* plots in this work, the *t* coordinate is given with respect to the Y subsystem.

The residuals on the satellite reflections improved when including the fourth-order parameter, and the refined value is more than five times its standard uncertainty [*x*_3_ = 0.0052 (5) sin(4π*t*_Y_) + 0.0035 (6) sin(8π*t*_Y_)]. Moreover, when including fourth-order terms, the range of Y—Y distances along the *c* axis (Fig. 4 ▸) match better with those of a commensurate superstructure refinement.

**Figure 5:**
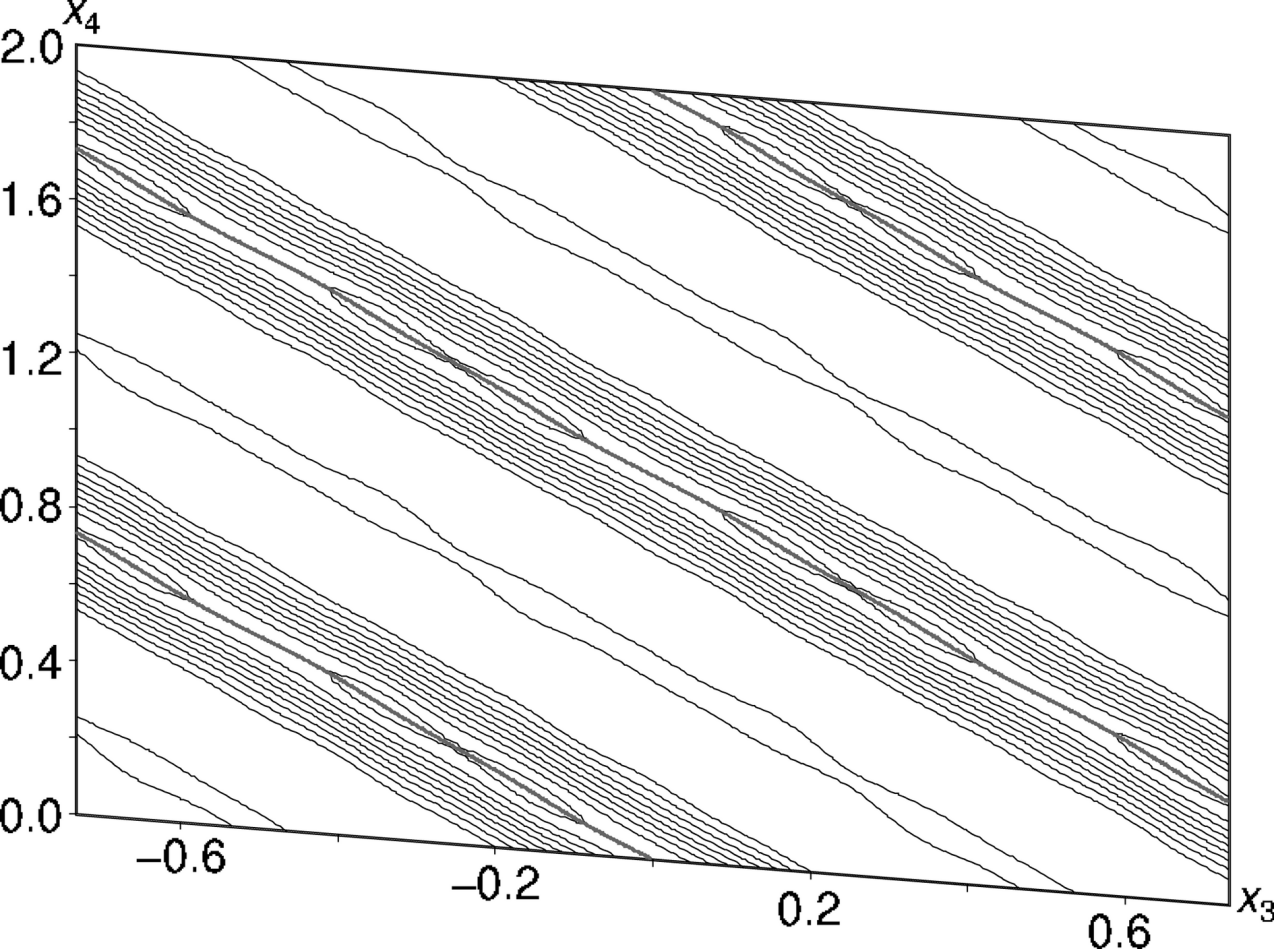
A 6 Å wide (*x*_3_, *x*_4_) section of superspace centered on the Y atom located at *x*_1_ = *x*_2_ = 0. The refined position of the Y atom is given by gray lines. Contours are drawn at the 20 e Å^−3^ level.

The absolute positional modulation is very subtle and barely visible in superspace sections (Fig. 5 ▸). Modulation of the ADPs did not improve the refinement and therefore the ADPs of Y were modeled as constant over internal space.

### 3.5. Modulation of the Os_4_B_4_ subsystem

Positional modulation of Os was modeled with harmonics up to the fourth order and that of B with harmonics up to the second order. The amplitudes of even higher harmonics refined to less than twice their standard uncertainties. As for Y, owing to symmetry, refinement of fourth-order Os harmonics corresponds to fewer parameters than one might expect. For first- and third-order harmonics, only displacement *perpendicular* to the *m*_〈110〉_ reflection planes is possible, corresponding to two parameters per harmonic (one amplitude and one phase) *versus* six parameters for an atom on the general position (two per spatial dimension). For the second-order and fourth-order harmonics, displacement is possible *parallel* to the *m*_〈110〉_ planes, corresponding to four parameters. Modulation of the Os ADPs was modeled with harmonics up to the second order. Modulation of the B ADPs did not improve the fit and was therefore omitted. An overview of the employed modulation parameters in given in Table 3 ▸.

**Figure 6:**
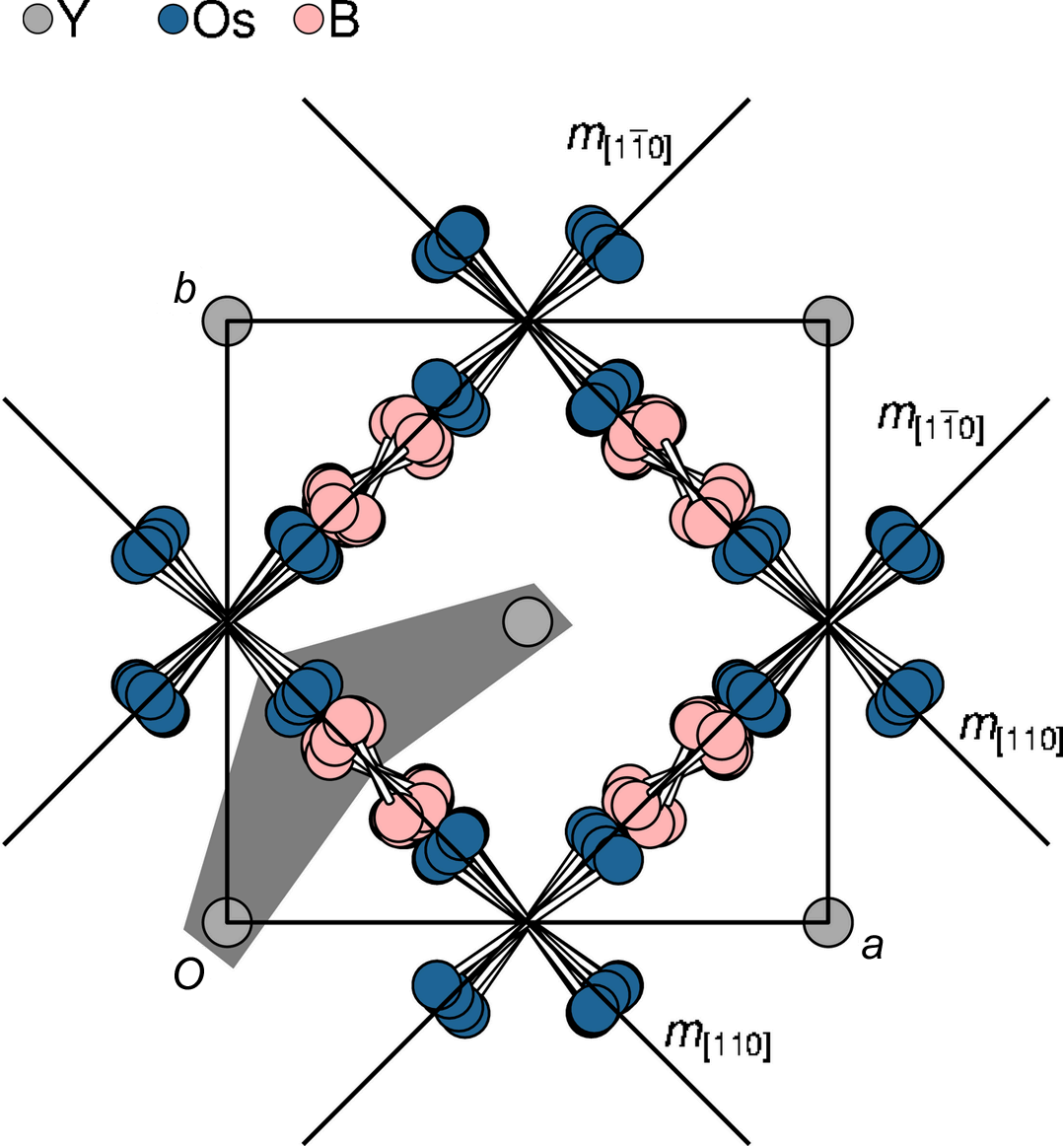
The modulated Y_*x*_Os_4_B_4_ structure viewed down [001]. In the Os columns, only the shared edges between adjacent tetrahedra are shown for clarity. The *m*_〈110〉_ planes on which the Os and B atoms are located in the basic structure are shown by the usual symbols. The gray background indicates the section shown later in Fig. 13.

**Figure 7:**
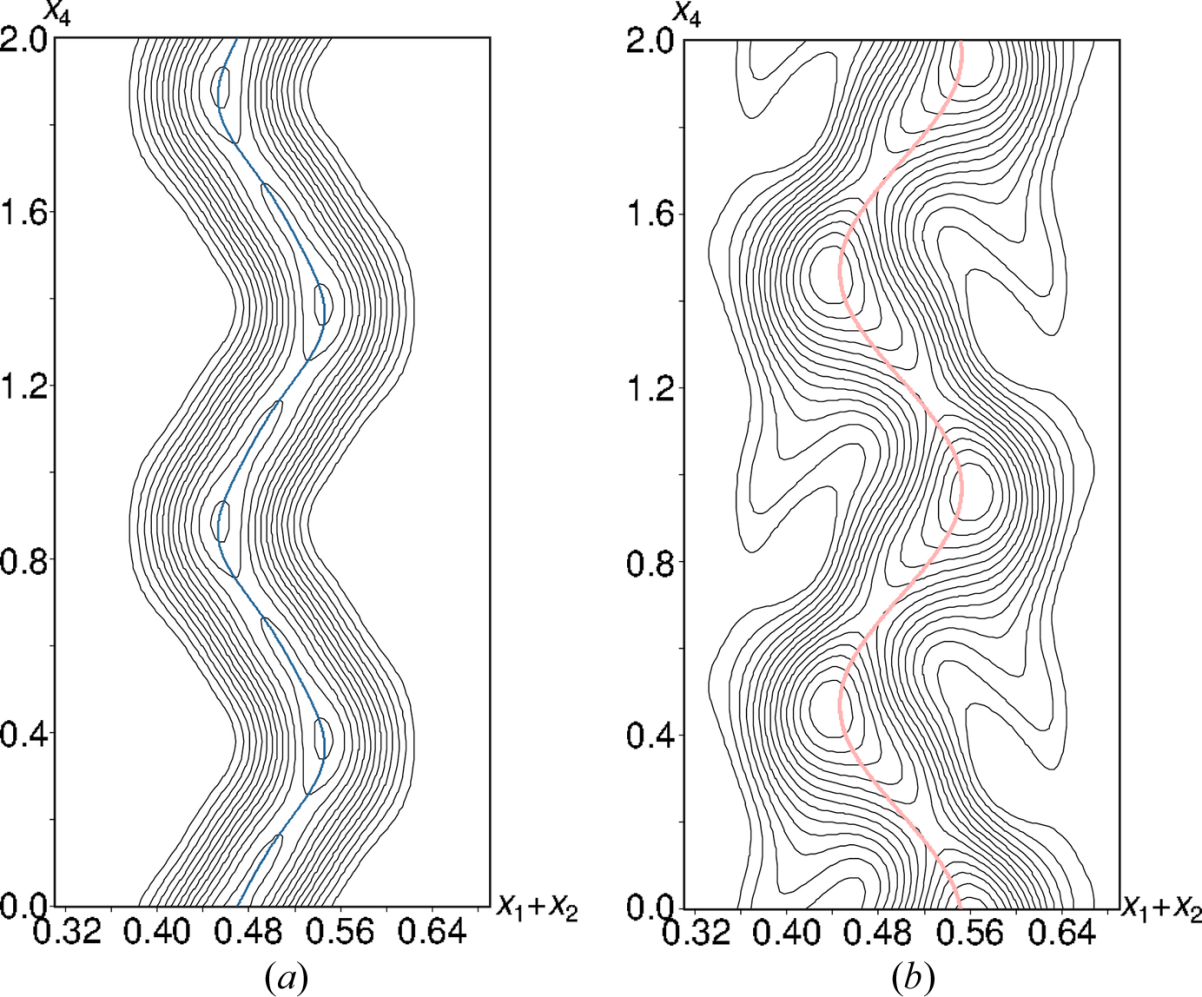
The 2 Å wide superspace sections in the (*x*_1_ + *x*_2_, *x*_4_) plane centered on (*a*) the Os and (*b*) the B atom, showing displacement away from the *m*_[110]_ reflection plane on which the Os and B atoms are located in the basic structure. Contours are drawn at levels of (*a*) 50 e Å^−3^ and (*b*) 2 e Å^−3^.

Displacement perpendicular to *m*_〈110〉_ is significantly more pronounced than in the plane (Fig. 6 ▸). No signs of dis­continuity of the modulation functions were observed in the superspace electron density (Fig. 7 ▸).

**Figure 8:**
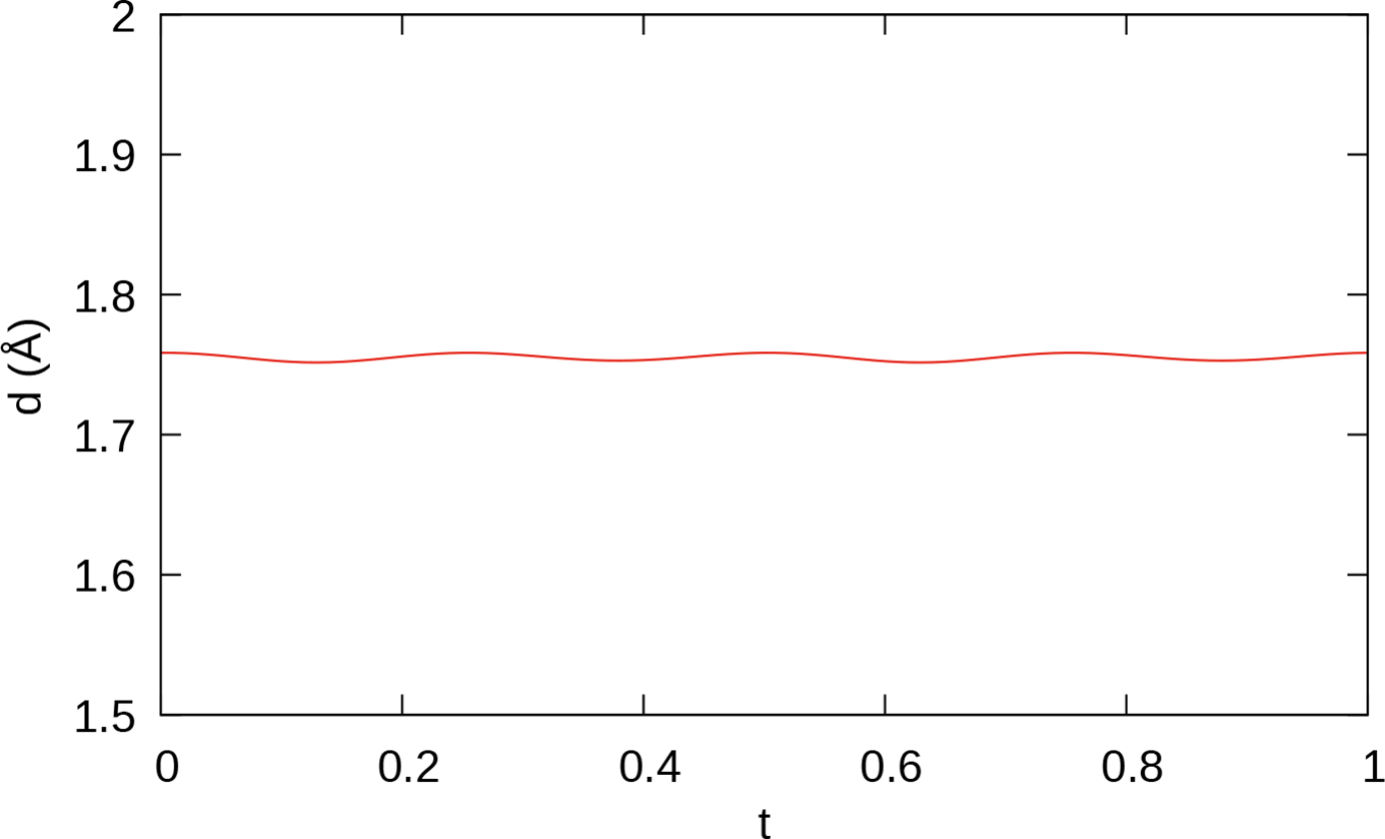
A *t* plot of B—B distances of the B_2_ dumbbells.

**Figure 9:**
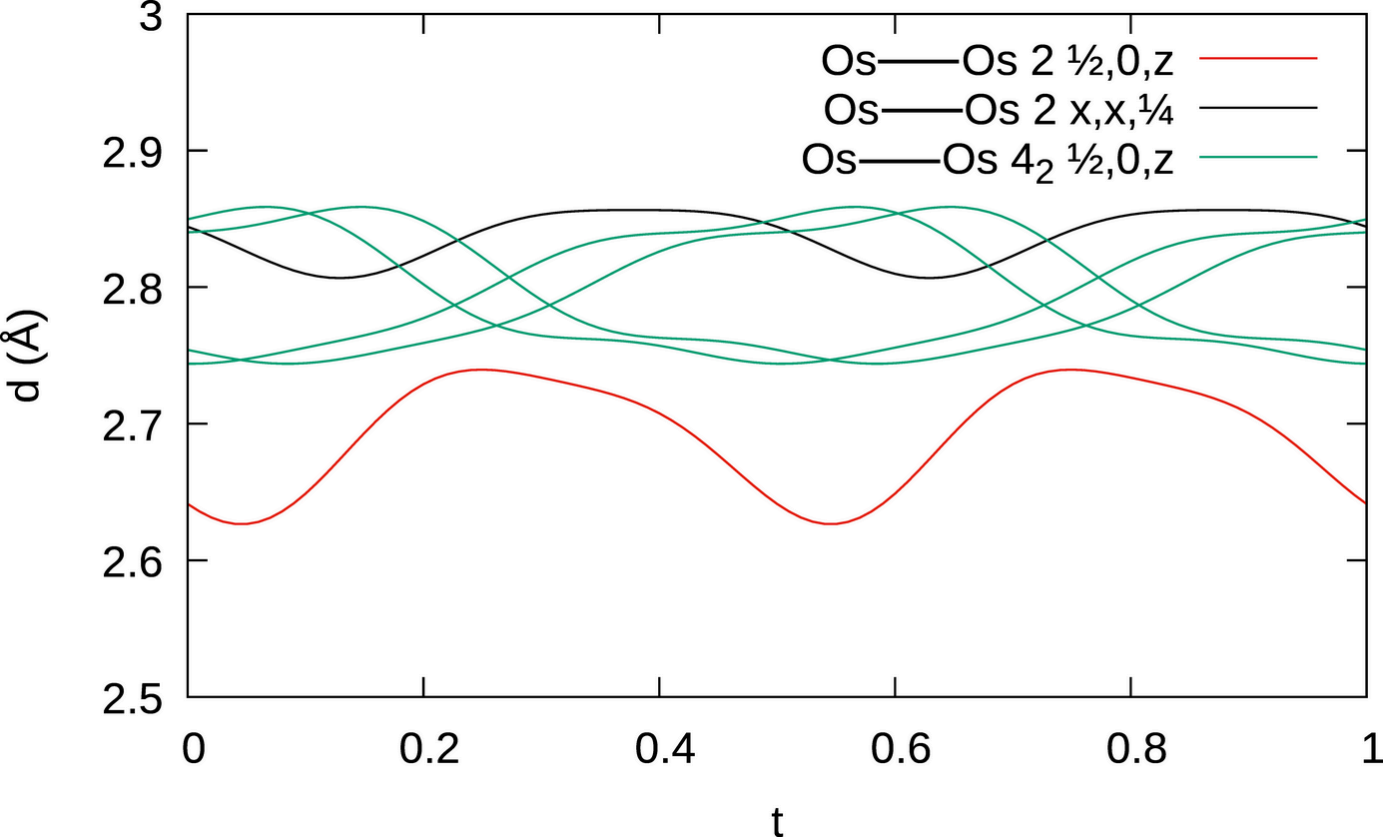
A *t* plot of Os—Os distances (red: edge-linked adjacent Os_4_ tetrahedra, green: remaining tetrahedron edges, black: bond connecting tetrahedra in different columns).

The modulation of the B_2_ dumbbells is best described as a rotation about the 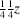 axis and symmetry equivalents (Fig. 6 ▸), leading to practically constant B—B bond lengths (Fig. 8 ▸). In contrast, the Os_4_ tetrahedra show significant distortions (Fig. 9 ▸). In particular, the shortest Os—Os bonds, which correspond to the shared edges between Os_4_ tetrahedra, vary from 2.627 (4) to 2.740 (4) Å (red curve in Fig. 9 ▸).

**Figure 10:**
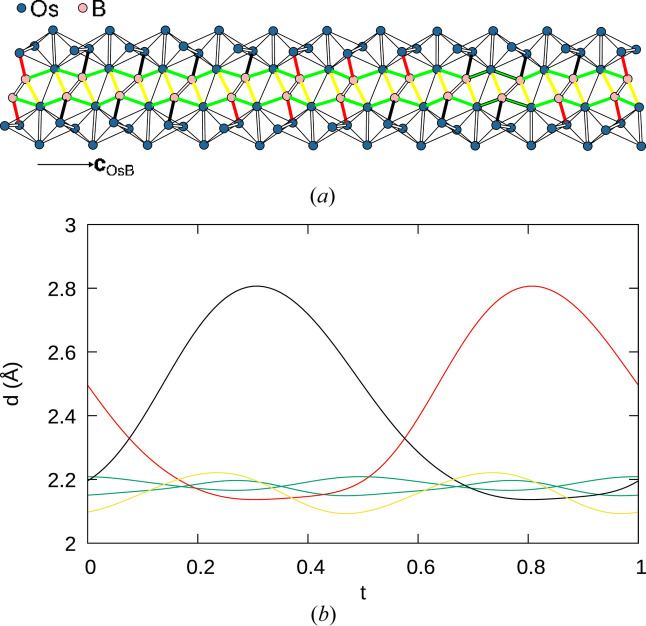
(*a*) Two adjacent Os columns connected by B_2_ dumbbells. Shared edges between Os_4_ tetrahedra are represented by thick lines and other Os—Os bonds by thin lines. (*b*) A *t* plot of Os—B distances. Color codes are discussed in the main text.

The B_2_ dumbbells connect adjacent Os columns. Fig. 10 ▸(*a*) shows two such adjacent Os columns and the connecting B_2_ dumbbells. The *t* dependence of the corresponding bond lengths is given in Fig. 10 ▸(*b*). Both figures use the same color coding of the Os—B bonds. In general, each B atom is co­ordinated to four Os atoms. Two bonds connect Os atoms in two adjacent Os_4_ tetrahedra in the same Os column (green in Fig. 10 ▸). A further bond is formed to the edge connecting these two tetrahedra. Owing to the positional modulation of the edge, the bond ‘switches’ in internal space between the two Os atoms of the edge (represented by red and black in Fig. 10 ▸). Finally, a fourth bond connects to the second Os column (yellow in Fig. 10 ▸).

### 3.6. Interactions of the subsystems

**Figure 11:**
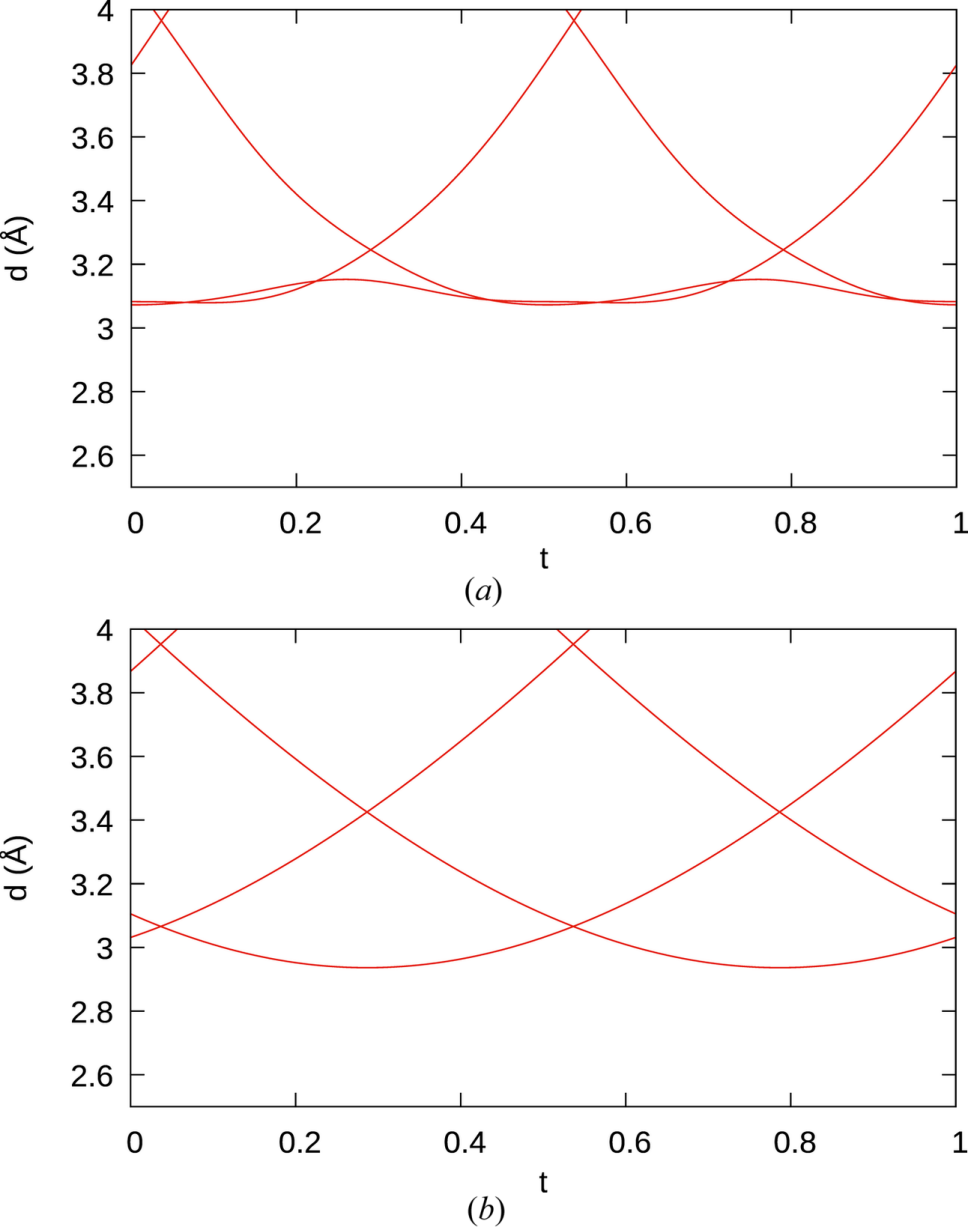
The *t* plots of the Os—Y distances in (*a*) actual Y_*x*_Os_4_B_4_ and (*b*) the hypothetical structure without positional modulation.

**Figure 12:**
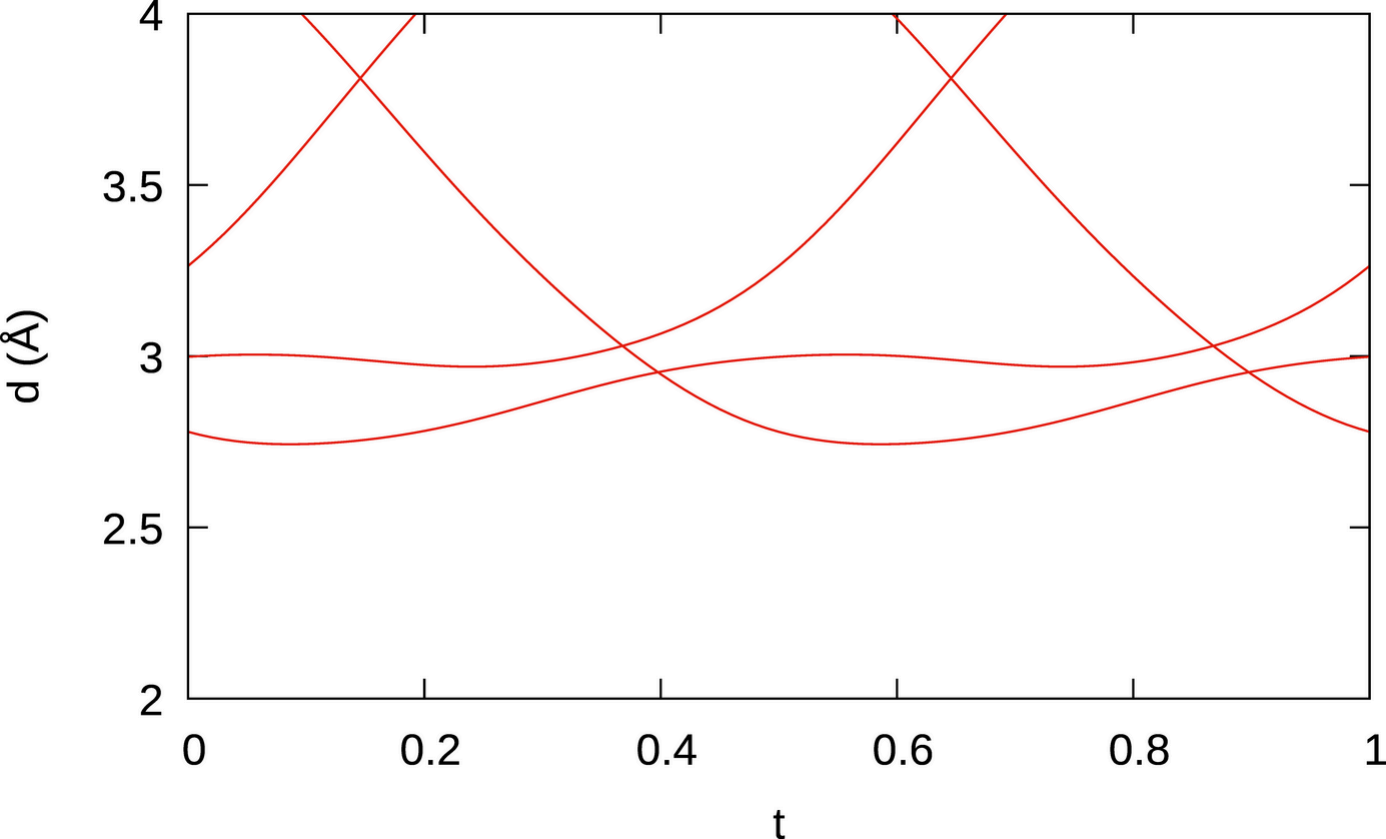
The *t* plots of the B—Y distances.

The pronounced modulation of the Os_4_B_4_ subsystem is certainly due to the interaction with the Y columns. Each Os and each B atom are connected to two Y columns (gray background in Fig. 6 ▸). Fig. 11 ▸ gives a comparison of the Os—Y distances in the modulated structure and the hypothetical structure without positional modulation. In the actual structure [Fig. 11 ▸(*a*)], Os is in general coordinated to two Y atoms with approximately equal distances. In small parts of the internal space there are three close Y atoms. There, as expected, the Os—Y distances are slightly longer. Coordination in the hypothetical non-modulated structure [Fig. 11 ▸(*b*)] is chemically less reasonable, with generally one very short (<3 Å) and one or two distant Y atoms. The coordination of the B atoms is similar, with regions of the internal space where B is close to two and other regions where it is close to three Y atoms (Fig. 12 ▸). Given the minute modulation of the Y subsystem, one can conclude that the Os_4_B_4_ subsystem adapts to the Y subsystem but not *vice versa*. This explains why an integration as a modulated Os_4_B_4_ structure was more successful than a classical integration as an incommensurate composite structure (see Section 3.1).

**Figure 13:**
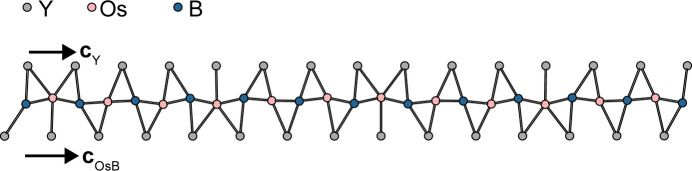
A column of Os and B atoms with adjacent Y atoms (marked by the gray background in Fig. 6), viewed down 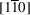. Y—Os and Y—B contacts are shown up to arbitrary cut offs of 3.3 Å and 3.1 Å, respectively.

The connectivity of Os/B columns to the two adjacent Y columns (gray background in Fig. 6 ▸) is shown in Fig. 13 ▸. Note that the average positions of the Y atoms in the two Y columns are translated by **c**_Y_/2, which corresponds to the pseudo-*I* centering of the basic structure of the Y subsystem.

## 4. Conclusion and outlook

Y_*x*_Os_4_B_4_ is an incommensurate composite where one subsystem (Os_4_B_4_) is significantly more modulated than the other (Y). Therefore, processing the raw data as if it were a regular modulated structure of the Os_4_B_4_ subsystem was preferred over a classical treatment as a composite system. Despite the issues of overlapping reflections, which are typical for aperiodic structures, a satisfying refinement was achieved, proving the robustness of single-crystal diffraction.

A detailed investigation of the physical properties of Y_*x*_Os_4_B_4_, including density functional theory calculations, will be published in an upcoming paper.

## Supplementary Material


wgToiDUSNfn

CCDC reference: 2389397

## Figures and Tables

**Table 1:** Data collection and refinement details for Y_1.161_Os_4_B_4_

Crystal data	
Chemical formula	B_4_Os_4_Y_1.161_
*M*_*r*_	907.3
Crystal system	Tetragonal
Temperature (K)	300
Radiation type	Mo *K*α
ρ_calc_ (g cm^−3^)	13.2532
μ (mm^−1^)	128.424
Crystal shape, color	Fragment, black
Crystal size (mm)	0.08 × 0.05 × 0.02
	
Data collection	
Diffractometer	Stoe STADIVARI
Absorption correction	Multi-scan
*T*_min_, *T*_max_	0.035, 0.187
No. of measured, independent and observed [*I* > 3σ(*I*)] reflections	16701, 1363, 1144
*R*_int_	0.0720
(sinθ/λ)_max_ (Å^−1^)	0.84
	
Refinement	
*R*_obs_, *wR*(*F*^2^)_obs_, *R*_all_, *wR*(*F*^2^)_all_	
All	0.0321, 0.1108, 0.0390, 0.1182
Main	0.0295, 0.1119, 0.0323, 0.1161
First order	0.0316, 0.1062, 0.0373, 0.1112
Second order	0.0455, 0.1173, 0.0722, 0.1329
No. of parameters	48
Δρ_max_, Δρ_min_ (e Å^−3^)	−3.73, 3.94
Extinction (Gaussian)	97 (17)

**Table 2:** Cell parameters and symmetry

	Subsystem 1 (Os_4_B_4_)	Subsystem 2 (Y)
Composition	B_4_Os_4_	Y
Superspace group	*P*4_2_/*ncm*(00σ_3_)00*ss*	*P*4_2_/*nmc*(00σ_3_)*s*0*s*0
Origin	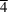	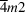 , shifted by 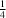 in the *x*_4_ direction
**W**	**I**	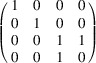
*a*, *c* (Å)	7.4495 (3), 4.0967 (2)	7.4495 (3), 3.5276 (2)
**q**	0.161335 (10)c*	0.861078 (10)c*
*V* (Å^3^)	227.347 (17)	195.764 (17)
*Z*	2	2

**Table 3:** Employed modulation parameters

	Position		ADP	
Atom	Maximum order	Number	Maximum order	Number
Os	4	12	2	12
B	2	6	–	–
Y	4	2	–	–
